# Interpretation of body-mounted accelerometry in flying animals and estimation of biomechanical power

**DOI:** 10.1098/rsif.2013.0404

**Published:** 2013-10-06

**Authors:** R. J. Spivey, C. M. Bishop

**Affiliations:** School of Biological Sciences, Bangor University, Bangor, Gwynedd LL57 2UW, UK

**Keywords:** birds and bats, root-mean-square acceleration, flapping flight, kinematic modelling, overall dynamic body acceleration, biomechanical power

## Abstract

An idealized energy fluctuation model of a bird's body undergoing horizontal flapping flight is developed, focusing on the biomechanical power discernible to a body-mounted accelerometer. Expressions for flight body power constructed from root mean square dynamic body accelerations and wingstroke frequency are derived from first principles and presented in dimensionally appropriate units. As wingstroke frequency increases, the model generally predicts a gradual transition in power from a linear to an asymptotically cubic relationship. However, the onset of this transition and the degree to which this occurs depends upon whether and how forward vibrations are exploited for temporary energy storage and retrieval. While this may vary considerably between species and individual birds, it is found that a quadrature phase arrangement is generally advantageous during level flight. Gravity-aligned vertical acceleration always enters into the calculation of body power, but, whenever forward acceleration becomes relevant, its contribution is subtractive. Several novel kinematic measures descriptive of flapping flight are postulated, offering fresh insights into the processes involved in airborne locomotion. The limitations of the model are briefly discussed, and departures from its predictions during ascending and descending flight evaluated. These findings highlight how body-mounted accelerometers can offer a valuable, insightful and non-invasive technique for investigating the flight of free-ranging birds and bats.

## 1. Introduction

Birds flap their wings in order to achieve weight support and locomotion [1–3]. Experiments using high-frame-rate video footage to monitor wing and body motions of birds or bats flying in wind tunnels have combined the findings with aerodynamic results and accelerometry to estimate overall energy expenditure during flight [4–6]. Such approaches have been experimentally valuable and theoretically illuminating, enabling the refinement of aerodynamic theory, but in studies involving free-ranging animals where trailing wires and heavy equipment cannot be used, ambulatory recording of body acceleration offers a viable and practical alternative. Accelerometry was initially restricted to wind tunnel work [7], but has now been miniaturized and demands relatively little electrical power. Commercially available micro-electromechanical transducers are now capable of faithfully recording high-frequency vibrations, offering a new means of studying the characteristics, kinematics and energetics of free-ranging avian flight [8,9] and, indeed, animal locomotion in general [10]. Because the long-term study of birds in the wild is becoming increasingly feasible, there is new scope to assess some of the difficult choices birds face during long-range migrations [11,12].

Traditional techniques for monitoring the metabolic rate or power input of free-ranging vertebrates include doubly labelled water [13,14] and heart rate, *f*_h_, derived from electrocardiography [15,16]. The latter approach offers good temporal resolution but has historically necessitated calibration of *f*_h_ against measurements of oxygen consumption, 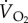. However, the direct translation of *f*_h_ to 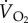 may now be possible for endotherms undergoing primary mode locomotion if augmented by knowledge of heart and body mass [17]. Accelerometry has a similar potential to monitor instantaneous biomechanical power output [18] during locomotion, and strong correlative relationships between body acceleration and 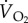 have been found in animals running on treadmills [18,19]. As one might expect, body accelerations during flight are generally elevated compared with other forms of locomotion [14,20–22]; however, a theoretical understanding of how body acceleration relates to the biomechanical power of flapping flight has not yet been elucidated. This study aims to address this by setting out a mathematical model that assists the interpretation of accelerometry data captured from birds during flight. Novel measures descriptive of flight kinematics, integral to this modelling, shall also be derived.

Instruments that log acceleration are, for practical reasons, generally attached to the torso of a flying animal. As wings are coupled to the body, this offers much promise as a non-invasive tool that can help estimate the biomechanical power (and indirectly or proportionally, the metabolic costs) associated with flight [18,19,23], with the potential to augment or replace existing methods [15,24]. Activity-related accelerations can be decomposed into the sum of dynamic and static accelerations which can be respectively derived by high- and low-pass filtering either in the time or the frequency domain. To date, biologists have found the dynamic component most informative with regard to correlations with energy expenditure. Two time-averaged measures of dynamic body acceleration (DBA) have been used when studying the energetics of animals [18,25]. Overall dynamic body acceleration (ODBA) is a running average of the *L*^1^-norm of the dynamic acceleration [10,18]. The variant using the *L*^2^-norm, which accurately encapsulates vectorial length, is known as vectorial dynamic body acceleration (VeDBA) [26,27]. An immediate difficulty with using any acceleration measure as a proxy for estimating biomechanical power in the absence of empirical calibration is that the fundamental units of acceleration, namely LT^−2^, are different both to those of power, ML^2^T^−3^, and those of mass-specific power, L^2^T^−3^. Overcoming this inevitably requires the development of some theoretical model descriptive of the biomechanics of bird flight with respect to body acceleration.

Under the hypothesis that DBA is closely related to overall metabolic costs, accelerometry has been successfully applied to a wide variety of other animals [10]. Consequently, it is not unreasonable to expect that a correlation could also exist between DBA and the biomechanical power directly discernible using a body-mounted accelerometer (*body power*) during steady horizontal flight and the metabolic rates of birds during flight (subject to the additional uncertainties of the value for the mechanochemical conversion efficiency of muscle [28]). Therefore, the ansatz is adopted here that the kinetics of the body should reflect the kinetics of the wings, thereby ultimately allowing the biomechanical costs incurred during avian locomotion to be estimated. This work primarily focuses on the interpretation of data from accelerometers attached to the body of a flying bird in the absence of additional information, a constraint demanding the development of some mathematical model to theoretically bridge the divide between body vibrations and overall biomechanical costs. The relationship between decomposed vertical and horizontal dynamic accelerations, and the various components of the energy associated with the body are investigated, lateral components being neglected due to the symmetrical beating of the wings. Birds must find ways of contending with the weight of gravity when airborne and flapping flight demands significant energy expenditure [1,3]. Motions of the body on the vertical axis differ from motions within the horizontal plane as they involve changes in gravitational potential. Thus, if the biomechanical power during flight is to be estimated solely from accelerometry, then it is essential to pay heed to the direction of gravity and hence also the absolute orientation of the transducer. The ultimate aim of this work is to derive estimates of acceleration-based biomechanical body power during flapping flight from first principles, potentially leading to the future interpretation of accelerometry from flying animals without the need for direct calibration. Novel statistical measures derived here may also be informative of flight kinematics, pertaining to energetically significant transitions in the wingstroke frequency, such as the relative phase and amplitude of forward and vertical body oscillations.

## 2. Developing a model

### 2.1. Preliminaries

Due to the pulsatile character of avian flight associated with the periodic contraction of powerful wing muscles, the energy associated with each wingstroke is delivered sporadically. Efficient flight confers evolutionary advantages, so the effort required by a bird to flap its wings is likely to achieve useful goals such as forward propulsion against aerodynamic drag, the countering of gravity, changes in velocity, ascent/descent and general manoeuvring. The mechanical energy of a bird will fluctuate in time and in still air there is a metabolic cost when the total energy increases. When it decreases, the dissipation of mechanical energy into the surrounding air is used to accomplish these various flight goals [29]. In the absence of gravity and an atmospheric medium, the mean mechanical power required by a vibrating but dissipationless mechanical system is precisely zero, because the total energy of the system is constant at all times. However, energy will necessarily be transformed or exchanged between different elements of the system within individual vibration cycles. Birds, on the other hand, remain airborne and sustain forward momentum despite air resistance by doing mechanical work which they never recover. Notwithstanding this, birds may be able to temporarily store and retrieve energy within individual wingbeat cycles in a similar manner to an idealized dissipationless system, so this should be considered when developing the model, as such a mechanism may provide significant flexibility to execute flight more efficiently under certain circumstances.

**Figure 1.**
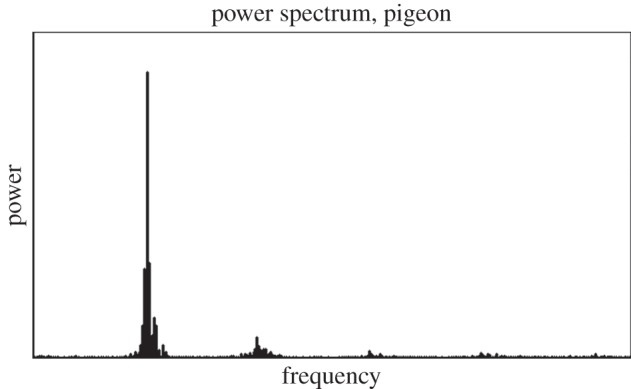
A typical power spectrum derived from the Fourier decomposition of about 10 s of vertical axis accelerometry data captured by the authors at 400 s s^−1^ from the body of a freely flying homing pigeon during horizontal, straightline flight while returning to a loft. The peak at the fundamental wingstroke frequency (at around 6.5 Hz) dominates over that of higher harmonics, suggesting that the acceleration on the bird's body is not only periodic but essentially sinusoidal.

We assume a triaxial accelerometer of negligible mass securely affixed to the body of a bird undergoing horizontal flight at a steady air speed. It has been known for several decades that, provided adequate consideration is given to harness design, accelerometers can be externally mounted to birds with negligible oscillation relative to the body [30]. Raw data from an accelerometer can be reoriented using mathematical transformations, an issue returned to later, so this analysis proceeds by taking the *z*-axis to be oppositely aligned to gravity and the *y*-axis to correspond to the direction of forward motion. An inertial frame of constant velocity comoving with the bird is considered. The majority of the power in the measured accelerations resides at the fundamental wingstroke frequency *f*, the angular frequency of the wingstrokes being *ω* = 2*πf*. This is a crucial assumption, backed up by the acceleration power spectrum obtained via accelerometry experimentally collected from a flying pigeon, as presented in figure 1. Because power is concentrated at the fundamental frequency, the oscillations of the body in the vertical axis can be accurately approximated by a sinusoid with maximum excursion *B* relative to the mean altitude. The vertical displacement *z* and vertical velocity 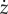 therefore vary as follows:2.1
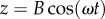
and2.2
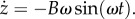

It is assumed that no energy is stored either elastically or as rotational kinetic energy, leaving the gravitational potential energy *U* = *m*_b_*gz*, the kinetic energy associated with the vertical axis 
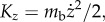
 and the kinetic energy associated with the forward axis 
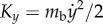
 to be considered. Here, *m*_b_ is the mass of the body of the bird, and *g* is the gravitational acceleration. The beating of both wings is assumed to always be in phase with one another. Because of the bilateral symmetry in birds, we neglect any kinetic energy associated with the *x*-axis.

The wings also have gravitational and kinetic energies (both vertical and horizontal). However, these are not directly estimable from a body-mounted accelerometer, so terms for these quantities are not explicitly included in the analysis. Instead, it is assumed that the activity detected by the accelerometer will, to a first approximation, be an attenuated reflection of the total biomechanical output of the bird. It is reasonable to think that energy associated with the wings can be subsumed into existing terms by constructive or destructive superposition. This follows from the fact that the addition of two arbitrarily scaled sinusoids of the same frequency, but different phase results in a rescaled sinusoid of the same frequency:2.3
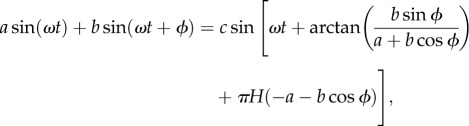
where 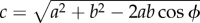 and *H* is the Heaviside step function in which *H*(*ξ*) = 1 if *ξ* ≥ 0, otherwise *H*(*ξ*) = 0. It can be seen that the resultant sinusoid will generally have an intermediate phase shift.

### 2.2. Relative phase between vertical and horizontal oscillations of the body

Vibrations on both axes will exhibit simple harmonic motion with both *K_y_* and *K_z_* varying at the same frequency. However, before an expression for the variation in *y* can be written as a sinusoid, there is a need to carefully consider its relative phase *θ* with respect to *z*. Even if the bird has considerable freedom to adjust the phase of its motion on the horizontal axis, arrangements that minimize the power required in order to sustain flight confer evolutionary advantages. Introducing a horizontal vibration amplitude *A*, the counterpart to *B* for the vertical axis, one can write2.4
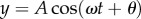
and2.5
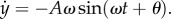


The convention is adopted that neither *A* nor *B* can be negative. The total energy associated with the bird's body, *E*_b_(*t*), is2.6
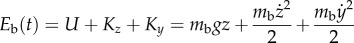
and2.7
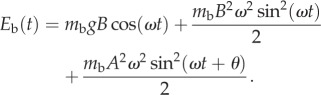


During each wingstroke, the flight muscles of the bird must supply energy when *E*_b_ is increasing. An important aspect of the present model is the assumption that when *E*_b_ decreases, energy is irrecoverably lost to the environment and exploited so as to achieve propulsion and weight support. This can be compared with aerodynamic models which assume the kinetic energy of the wings is never recovered [31,32]. The mean power associated with the body, 〈*P*_b_〉, can then be determined according to2.8
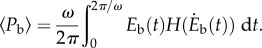


Consider, for now, the case in which *A* = 0. One then has *K_y_* = 0 and *E*_b_ = *U* + *K_z_*. Gravitational potential energy attains a minimum at *t* = *π*/*ω* when *z* = −*B* and a maximum at *t* = 0 when *z* = *B*. At both these times, *K_z_* reaches its minimum of zero. Because sin^2^*ωt* = (1 − cos 2*ωt*)/2, the vertical kinetic energy varies sinusoidally at twice the frequency of the gravitational energy. Thus, *E*_b_(*t* = *π*/*ω*) is always a minimum. Although *U* is always a maximum at *t* = 0, it is possible that *E*_b_ is not a maximum at that time if *K_z_* thereafter increases more rapidly than *U* decreases. This possibility is apparent upon inspection of the time derivatives of *E*_b_, the roots of which correspond to stationary points:2.9
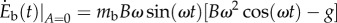
and2.10
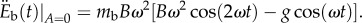
Owing to the sin(*ωt*) term, stationary points exist at *t* = 0 and *t* = *π*/*ω*. At *t* = *π*/*ω*, one has 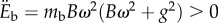, so this stationary point is always a minimum. However, 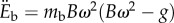 for the stationary point at *t* = 0, which corresponds to a maximum when *ω*^2^ < *g*/*B* and a minimum when *ω*^2^ > *g*/*B*. If both these are minima, additional stationary points occur when cos(*ωt*) = *g*/*B*ω**^2^. Using cos(2*ωt*) = 2*g*^2^/*B*^2^*ω*^4^ − 1, one then finds that at those times 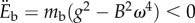, confirming that these points correspond to maxima of *E*_b_.

Therefore, when *ω*^2^ < *g*/*B*, the mean body power is simply 〈*P*_b_〉 = (*ω*/2*π*) (*E*_b_^max^ − *E*_b_^min^), where 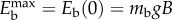 and 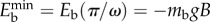. However, when *ω* > *g*/*B*, then due to the existence of a new maximum in *E*_b_ at *t* ≠ 0, 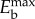 will exceed *E*_b_(0), and the mean power will inevitably rise. During intense flight, there will be a high wingstroke frequency, and for each wingstroke the variation in kinetic energy will increase and the variation in gravitational energy will decrease, allowing the variations in kinetic energy to become dominant. However, if it were possible to temporarily store some of the vertical kinetic energy as horizontal kinetic energy, and retrieve it later in the wingstroke cycle, then this elevated maximum in *E*_b_ could be avoided, and the concomitant increase in power is eliminated.

The transition between the two regimes occurs at *g* = *B*ω**^2^, corresponding to the peak gravitational energy *U*^max^ = *m*_b_*gB* being equal to twice the peak vertical kinetic energy 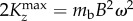. Clearly, if the total kinetic energy *K_yz_* = *K_y_* + *K_z_* did not fluctuate at all, then *K_z_* could be arbitrarily large without incurring any additional increase in mean power. The total kinetic energy is2.11
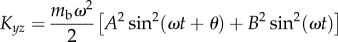
and2.12
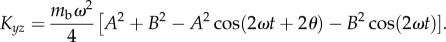
If *K_yz_* is constant, then its derivative2.13
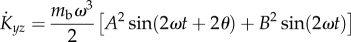
must be zero at all times. It is apparent from this expression and (2.3) that *K_yz_* varies sinusoidally. The amplitude of these fluctuations vanishes when2.14
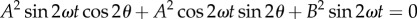
and2.15
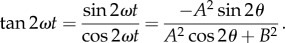
Because *θ* is constant, this would demand that *t* is also constant. However, *K_yz_* can be zero, if the sin 2*ωt* and the cos 2*ωt* terms are simultaneously zero. One then finds that the cos 2*ωt* term vanishes if sin 2*θ* = 0, which is satisfied when 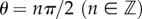. The sin 2*ωt* term is zero when *A*^2^ cos 2*θ* + *B*^2^ = 0, which yields *θ* = arccos(−*B*^2^/*A*^2^)/2. When *θ* = *n*π**, one has cos 2*θ* = 1 which must be rejected as it predicts *A*^2^ = −*B*^2^. However, *θ* = (*n* ± 1/2)*π* yields *A*^2^ = *B*^2^, which is acceptable. Therefore, for *K_yz_* to remain constant requires *A* = *B* and *θ* = ±*π*/2. This can be seen in the following2.16
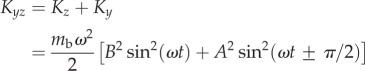
and2.17
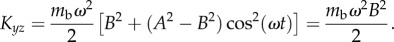
A relative phase shift of ±*π*/2 between the horizontal and vertical axes corresponds to what is commonly termed a quadrature phase arrangement. Only in this circumstance does it hold that *K_y_* is a maximum when *K_z_* is a minimum, and vice versa. This maximizes the potential for shuttling energy back and forth between the two axes, a useful property that the bird might be able to exploit to decrease its mean power. Although *K_y_* will be maximal at *t* = 0 and *t* = *π*/*ω*, because these maxima are equal, they have no effect on the difference *E*_b_(*t* = 0) − *E*_b_(*t* = *π*/*ω*). In fact, this holds for any value of *θ* because sin^2^*x* = sin^2^(*x* + *π*), but *K_yz_* is constant only when *K_y_* and *K_z_* are in antiphase. As the kinetic energies vary at double the fundamental wingstroke frequency, this occurs when *θ* =±*π*/2.

While variations in *K_yz_* can be completely eliminated, it may not be necessary for the bird to do so because, as will be shown, the bird can in some cases also minimize power when *A*/*B* < 1 with excessive fluctuations in *K_z_* being completely tamed by smaller fluctuations in *K_y_*. This may be preferable as it reduces extraneous energy losses and ameliorates the vibrations transmitted to the head of the bird, which might otherwise make flight an unnecessarily uncomfortable experience compromising visual acuity [33]. Note also that if *A*/*B* > 1, then fluctuations in *K_y_* may not be adequately absorbed by fluctuations in *K_z_*.

Because birds are expected to have a maximum forward velocity at the end of the downbeat, the phase that makes 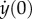 maximal is chosen (*θ* = −*π*/2). Hence, *θ* can be eliminated from the expressions for *y* and 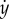 by writing2.18
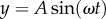
and2.19
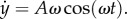
The upbeat commences at *t* = 0 when 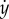 is maximal. The body is then at its maximum height above the ground. The body and the wings are in antiphase on the vertical axis, and the same should also be approximately true of the forward axis. To summarize the findings of this section, power reduction is possible only when *g*/*B*ω**^2^ < 1 and is best achieved by a quadrature phase arrangement.

### 2.3. Quadrature phase flight

Unless expressly stated otherwise, the analysis now proceeds by assuming quadrature phase applies. In order to assess the biomechanical power in the body, one is interested in determining the maxima and minima (stationary points) of the body energy, necessitating looking for roots of the first-time derivative and inspecting their signs by taking the second-time derivative. Both *K_yz_* and *U* vary sinusoidally but because they do not vary at the same frequency their sum is not a simple sinusoid, demanding that calculus be used. Body energy now reads2.20
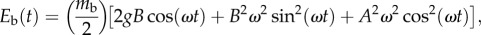
and the first derivative is2.21
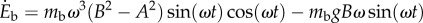
and2.22
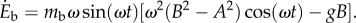
Stationary points exist when either cos(*ωt*) = *gB*/*ω*^2^(*B*^2^ − *A*^2^) or sin(*ωt*) = 0. The first condition has real solutions only if *ω*^2^ ≥ *gB*/|*B*^2^ − *A*^2^|. When *ω*^2^ > *g*/*B*, the smallest value of *A* that prevents a maximum in *E*_b_ from arising anywhere but at *t* = 0 occurs when cos(*ωt*) = 1, from which the smallest value of *A* that minimizes the power can be determined. If this is an overriding consideration with the need to maintain visual acuity a secondary concern, this value of *A* would seem to be optimal:2.23
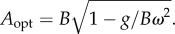


Note that *A*_opt_ must be real (because *ω*^2^ > *g*/*B*) and because 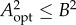 at all times, birds have no need to fly with *A* > *B.* Moreover, for given values of *A* and *B*, the optimum angular frequency comes to 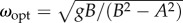 which exceeds the critical frequency 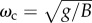 above which power can be reduced if *A* ≠ 0. The optimum ratio of *A*/*B* ensures fluctuations in *K_y_* are sufficiently large to avoid excessive fluctuations in *K_z_*, its value depending only on the ratio *ω*/*ω*_c_:2.24
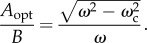


**Figure 2.**
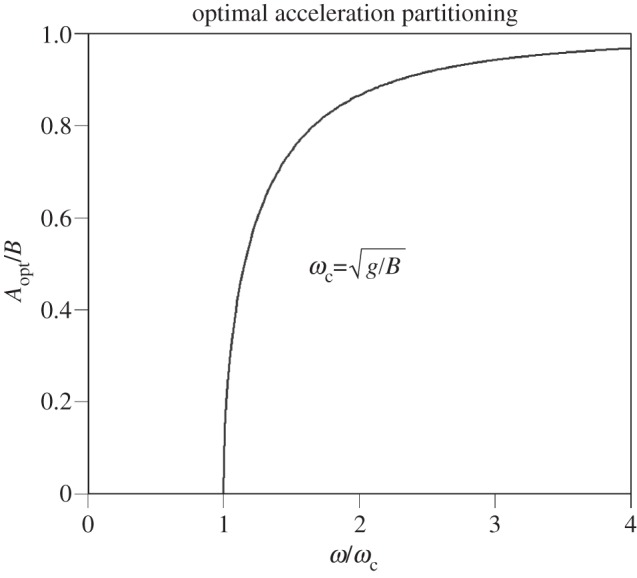
The optimum value of *A*/*B*, the ratio of the horizontal and vertical vibration amplitudes, at which body power is minimized and dynamic acceleration on the forward axis attains its smallest acceptable value, see equation (2.24).

This is plotted in figure 2. The second derivative of the body energy is2.25
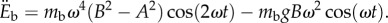

The condition that 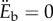 at *t* = 0 yields *A* = *A*_opt_, showing that the maximum at *t* = 0 is then also a stationary point of inflection. Now, consider the stationary points that arise when cos(*ωt*) = *gB*/*ω*^2^(*B*^2^ − *A*^2^). Using the identity cos(2*ωt*) = 2cos^2^(*ωt*) − 1, one finds that2.26
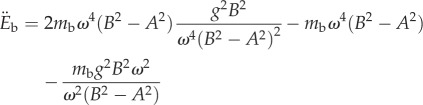
and2.27
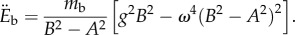
If *A*^2^ < *B*^2^, the sign of 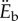 must be negative if *gB* < *ω*^2^(*B*^2^ − *A*^2^). This satisfies the condition required for the stationary points to exist, establishing that those corresponding to cos(*ωt*) = *gB*/*ω*^2^(*B*^2^ − *A*^2^) < 1 must be maxima if *A*^2^ < *B*^2^. Considering the alternative situation in which *A*^2^ > *B*^2^, the sign of 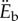 must be positive if *gB* < *ω*^2^(*A*^2^ − *B*^2^). Hence, the stationary points at which cos(*ωt*) = *gB*/*ω*^2^(*B*^2^ − *A*^2^) < 1 must then correspond to minima.

Note that the stationary point in *E*_b_ at *t* = *π*/*ω* has second derivative2.28
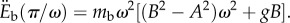


This is negative if *gB* < *ω*^2^(*A*^2^ − *B*^2^) so that when *A*^2^ > *B*^2^, which gives rise to stationary points at cos(*ωt*) = −*gB*/*ω*^2^(*A*^2^ − *B*^2^), the stationary point at *t* = *π*/*ω* becomes a maximum. This is a point of inflection when *gB* = *ω*^2^(*A*^2^ − *B*^2^) corresponding to the maximum value of *A* at which power is minimized:2.29
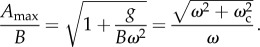


**Figure 3.**
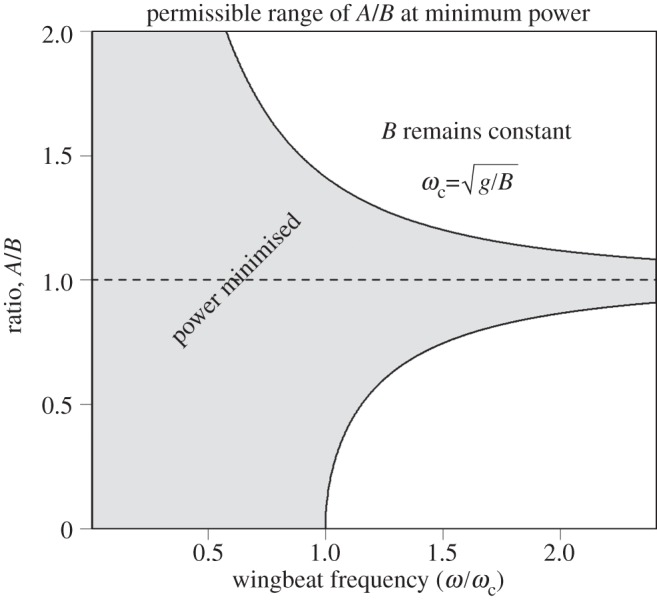
Body power is minimized providing the ratio *A*/*B* falls within the interval 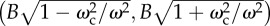 if *ω* > *ω*_c_ or 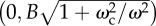 if *ω* ≤ *ω*_c_. The upper and lower limits to *A*/*B* each approach unity as *ω*/*ω*_c_ → *∞* (see text).

This has a similar form to the earlier expression 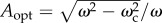. The bird has minimized its mean body power if *A*_opt_ ≤ *A* ≤ *A*_max_ or equivalently if *ω*^2^ < *gB*/|*B*^2^ − *A*^2^|. When this condition is satisfied and *ω*^2^ > *g*/*B*, flapping flight demands less power than when forward axis vibrations are absent altogether (*A* = 0). For a fixed value of *B*, this is illustrated in figure 3.

## 3. Estimation of body power

**Figure 4.**
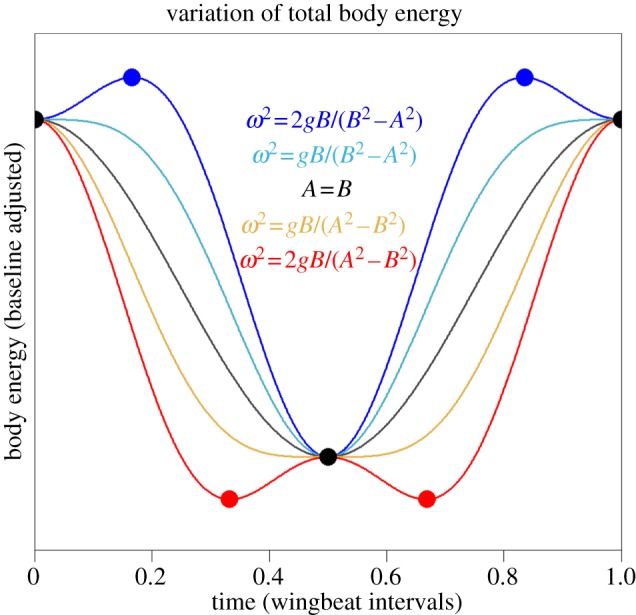
Variation of body energy assuming motions on the forward and vertical axes are in quadrature phase (see text). In this model, the bird provides the energy required for the traces to increase with time but does not recover that energy when it declines. Thus, differences in energy between the stationary points (marked by dots) allow the calculation of mean power. There are two limiting cases: (i) *ω*^2^ = *gB*/(*B*^2^ − *A*^2^), when *B*^2^ > *A*^2^, and (ii) *ω*^2^ = *gB*/(*A*^2^ − *B*^2^), when *A*^2^ > *B*^2^ (both plotted) delineating three modes of flight. The case *A* = *B* bisects the range in which the mean power attains a minimum, which becomes narrower as wingstroke frequency increases (figure 3). Outside this range, stationary points appear at times intermediate between the mid-point and the start/end points of a wingstroke cycle (at *t* = t*, upper and lower traces provide examples). (Online version in colour.)

The calculation of mean body power here involves the integration of *E*_b_ only as it increases according to (2.8). This can be easily accomplished by subtracting the minima of *E*_b_ from successive maxima of *E*_b_. If *t* = 0 at *t* = *t*_0_, *t* = *π*/*ω* at *t* = *t*_1_ and *t* = ±arccos[*gB*/*ω*^2^(*B*^2^ − *A*^2^)]/*ω* at *t* = t* then only these times need be considered in order to determine the minima and maxima of the body energy variation. With reference to figure 4, it can be seen that there are three distinct cases to consider with stationary points located at the following times:

case 1: *t* = *t*_0_ and *t* = *t*_1_: {*gB* > *ω*^2^|*B*^2^−*A*^2^|},
case 2: *t* = *t*_0_, *t* = *t*_1_ and *t* = t* {*gB* < *ω*^2^(*B*^2^−*A*^2^); *A*^2^ < *B*^2^; *E*_b_(t*) > *E*_b_(*t*_0_)} and
case 3: *t* = *t*_0_, *t* = *t*_1_ and *t* = t* {*gB* < *ω*^2^(*A*^2^−*B*^2^); *A*^2^ > *B*^2^; *E*_b_(t*) < *E*_b_(*t*_1_)}.

The body energy at *t*_0_ and *t*_1_ is simply3.1
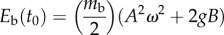
and3.2
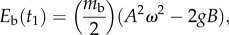
When *t* = t*, sin^2^(ωt*) is obtained as 1 − cos^2^(ωt*):3.3
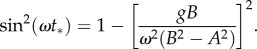


After some algebra, the total energy of the body when *t* = t* is found to be3.4
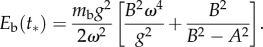


### 3.1. Case 1

This case corresponds to the shaded region in figure 3 and the central traces of figure 4 for which the calculation of mean power is particularly simple:3.5
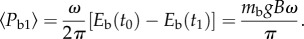


### 3.2. Case 2

This case corresponds to the zone below the shaded region in figure 3 (see also the uppermost trace in figure 4). The stationary point at *t*_0_ is now a minimum and two new maxima arise at *t* = t*. Recalling that in this case, *B*^2^ > *A*^2^, the power is3.6
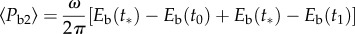
and3.7
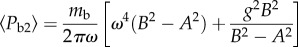


### 3.3. Case 3

This case corresponds to the zone above the shaded region in figure 3 (see also the lowermost trace in figure 4). The stationary point at *t*_1_ is now a maximum and new minima arise at *t* = t*. One has *A*^2^ > *B*^2^, and the power now comes to3.8
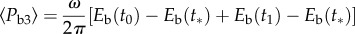
and3.9
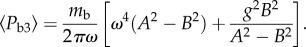
The poles appearing in the expressions for 〈*P*_b2_〉 and 〈*P*_b3_〉 as *A* → *B* are avoided because when *ω*^2^ > *gB*/|*B*^2^ − *A*^2^| one has *A*^2^ ≠ *B*^2^. It is apparent that the results for cases 2 and 3 are equivalent but for the reversal of sign in the *B*^2^ − *A*^2^ terms. Therefore, it would be acceptable to take the modulus of either expression without expressly checking whether *A*^2^ > *B*^2^. In the limiting cases where *gB* = *ω*^2^|*B*^2^ − *A*^2^|, the prediction of case 1 coincides with that of case 2 or 3. For instance, when 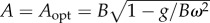 then *B*^2^ − *A*^2^ = *gB*/*ω*^2^ and it can be seen that 〈*P*_b2_〉 reduces to 〈*P*_b1_〉:3.10
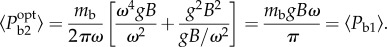


The importance of correctly distinguishing between case 1 and cases 2 and 3 is stressed, because 〈*P*_b2_〉 and 〈*P*_b3_〉 overpredict the true power when *gB* > *ω*^2^|*B*^2^ − *A*^2^| and 〈*P*_b1_〉 underpredicts the true power when *gB* < *ω*^2^|*B*^2^ − *A*^2^|.

### 3.4. Deriving body power using data from accelerometers

The root mean square (or r.m.s.) value of a quantity is a measure commonly used in physics and engineering and can lend itself to the description of DBA (table 1). As such, it offers an alternative to ODBA and VeDBA. The r.m.s. value of a discrete set of *N* dynamic single axis accelerations *a*_dyn_ is 
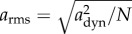
, and that of a simple sinusoid such as *ψ* = *β* sin(*t*) is3.11
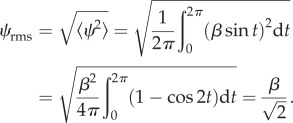

**Table 1.** Variables used in this study.

variable	units	description
***a***	m s^−2^	acceleration vector
*a*_rms_	m s^−2^	r.m.s. acceleration
*a*_dyn_	m s^−2^	dynamic acceleration
*A*	m	relative forward displacement amplitude
*A*_opt_	m	smallest value of *A* that minimizes power
*B*	m	relative vertical displacement amplitude
*E*_b_	J	body energy
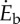	J s^−1^	first-time derivative of body energy
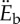	J s^−2^	second-time derivative of body energy
*f*	Hz	wingstroke frequency
*f*_h_	beats min^−1^	heart-rate
*g*	m s^−2^	Earth's gravitational acceleration
*K_y_*	J	relative forward kinetic energy
*K_z_*	J	relative vertical kinetic energy
*K_yz_*	J	total kinetic energy
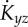	J	first-time derivative of total kinetic energy
L	m	dimension of length
M	kg	dimension of mass
*m*_b_	kg	body mass
ODBA	m s^−2^	overall dynamic body acceleration
*ω*	rad s^−1^	wingstroke angular frequency
*ω*_opt_	rad s^−1^	optimal value of *ω*
*ω*_c_	rad s^−1^	first critical value of *ω*
*ω*_0_	rad s^−1^	second critical value of *ω*
*P*_b_	W	biomechanical body power
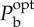	W	optimal biomechanical body power
*ϕ*	rad	rotation angle
T	s	dimension of time
*t*	s	time
*θ*	rad	relative phase angle
*U*	J	gravitational potential energy
VeDBA	m s^−2^	vectorial dynamic body acceleration
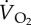	ml min^−1^	oxygen consumption rate
*y*	m	relative forward displacement
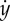	ms^−1^	relative forward velocity
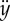	m s^−2^	forward acceleration
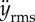	m s^−2^	r.m.s. forward acceleration
*z*	m	relative vertical displacement
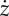	m s^−1^	relative vertical velocity
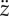	m s^−2^	vertical acceleration
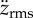	m s^−2^	r.m.s. vertical acceleration

Static and dynamic accelerations can be respectively derived from raw acceleration data using low-pass and high-pass filtering techniques. While the static acceleration is useful in determining the vertical, gravity-aligned axis, there are many circumstances where the direction of the forward axis is more ambiguous. However, birds will generally adjust the roll of their bodies during flight so that the static acceleration vector remains dorsally aligned. It is therefore likely that the r.m.s. value of the dynamic sway, 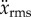, will be appreciably smaller than the r.m.s. value of the dynamic surge, 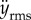. In principle, this allows for reorientation of accelerometry data during post-processing by application of a rotation matrix whose components can be inferred by analysis of the data. A method for achieving reorientation is outlined in appendix A. Estimates of the dynamic surge, 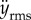, and the dynamic heave, 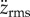, are readily obtained after high-pass filtering of the reoriented acceleration components. These relate to *ω*, *A* and *B* as follows:3.12
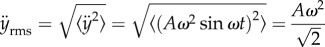
and3.13
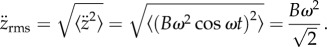
Hence, body power can be directly evaluated from r.m.s. heave and surge:3.14
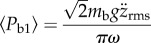
and3.15
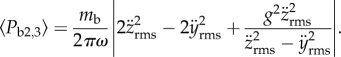
These expressions conveniently obviate the need to double integrate acceleration data in order to obtain the values of *A* and *B* directly, which is generally challenging due to the baseline drift introduced when integrating. However, *A* and *B* each feature in the true-or-false test *gB* < *ω*^2^|*B*^2^ − *A*^2^| that determines which expression for power is valid. Therefore, a reformulation of the discriminant is also desirable, and because 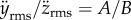 it follows that3.16
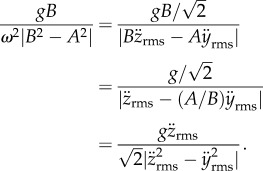


It is therefore possible to determine, without knowledge of either *A* or *B*, nor indeed *ω*, the regime in which the bird is flying. 〈*P*_b1_〉 should be used when 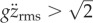 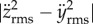, otherwise 〈*P*_b2,3_〉 is applicable.

The critical frequency ratio, *ω*/*ω*_c_, can similarly be translated using (3.13) and the knowledge that 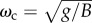 3.17
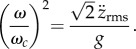


It is also possible to express *ω*/*ω*_opt_ using only r.m.s. accelerations:3.18
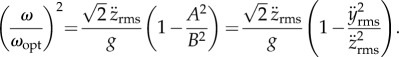


### 3.5. Relative body power

**Figure 5.**
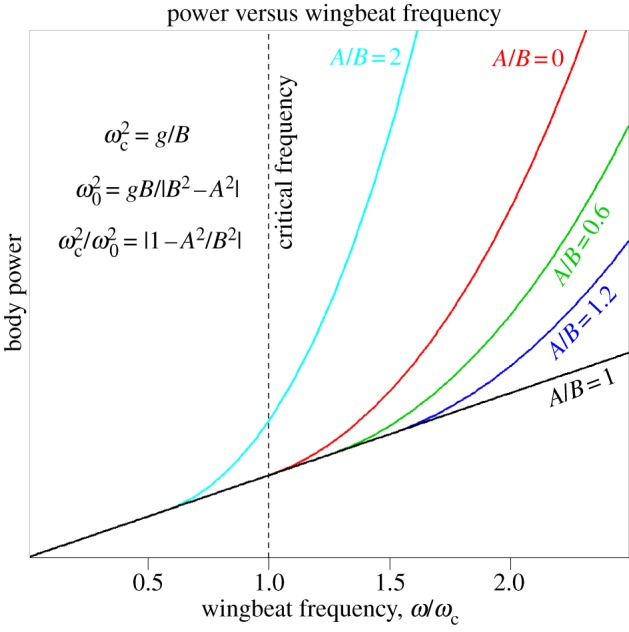
Above a threshold frequency that depends on the ratio *A*/*B*, when *A* ≠ *B* an asymptotically cubic response is inevitable at high frequencies. However, body power is always minimized when *A* = *B*. See equations (3.5), (3.7), (3.9) and (3.22). (online version in colour.)

According to this model, body power retains linearity with wingstroke frequency until, and as illustrated in figure 5, the angular frequency exceeds3.19
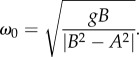

This can also be conveniently gauged from r.m.s. accelerations alone because3.20
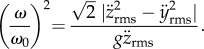


The expressions for 〈*P*_b2_〉 and 〈*P*_b3_〉 can be recast as3.21
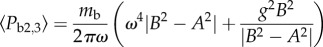
and3.22
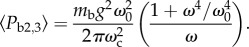
It is now apparent that when 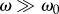, the response becomes asymptotically cubic at higher wingstroke frequencies, i.e. 〈*P*_b2,3_〉 ∝*ω*^3^. However, at lower wingstroke frequencies, flight proceeds within the linear regime of (3.5). For case 1, it is possible to write 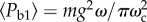, allowing the ratio of 〈*P*_b1_〉/〈*P*_b2,3_〉 to be expressed in a particularly simple form3.23
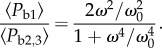


**Figure 6.**
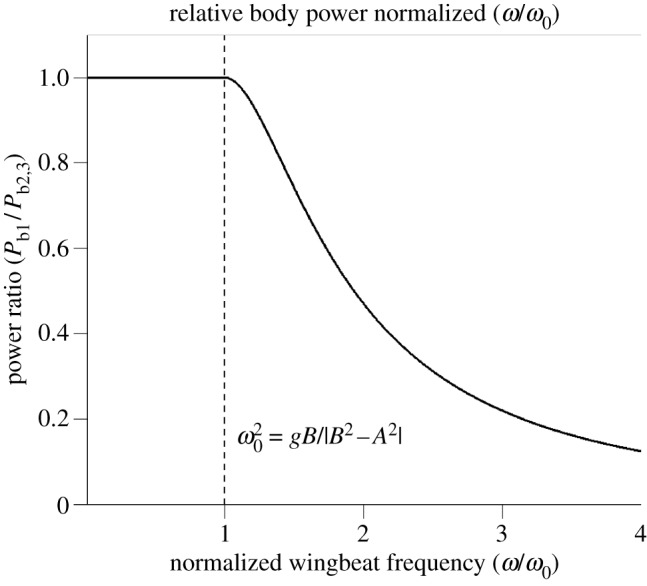
The ratio 〈*P*_b1_〉/〈*P*_b2,3_〉 as a function of normalized wingstroke frequency *ω*/*ω*_0_. When *ω* < *ω*_0_, forward vibrations are incapable of reducing the mean body power. Note that 〈*P*_b1_〉 never exceeds 〈*P*_b2,3_〉.

The response is plotted in figure 6 and can be compared with figure 5 where the wingstroke frequency is expressed in units of *ω*/*ω*_c_. If *ω* < *ω*_0_, then the expression for 〈*P*_b2,3_〉 is not physically meaningful, and the mean power is always given by 〈*P*_b1_〉. When *ω* > *ω*_0_, power can always be reduced, because the ratio 〈*P*_b2,3_〉/〈*P*_b1_〉 then exceeds unity. Furthermore, it grows without limit as *ω* → ∞.

Power is always minimized when *A* = *B* but it is interesting to know how the ratio 〈*P*_b2,3_〉/〈*P*_b1_〉 grows for other values of *A*/*B* when *ω* > *ω*_0_. First, note that *ω*_0_ is related to *ω*_c_ according to3.24
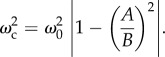


**Figure 7.**
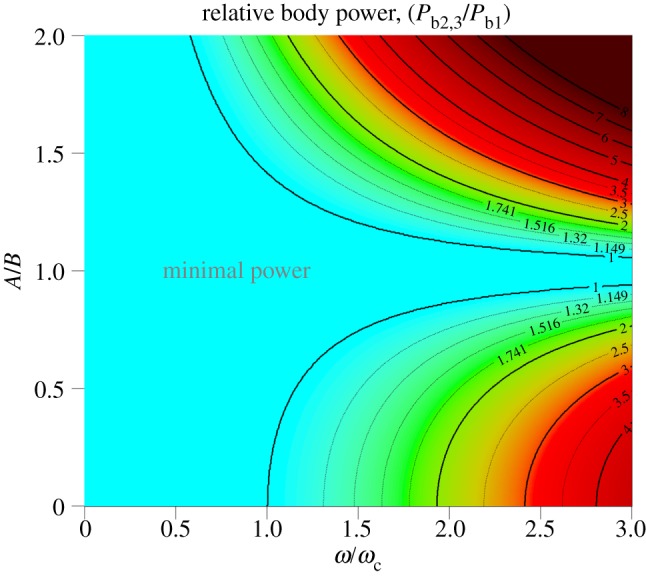
The relative cost of flapping flight normalized to that possible when *A* = *B*. For example, at a constant wingstroke frequency *ω*/*ω*_c_ = 2, flight costs more than double when 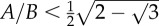 or 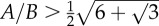. (Online version in colour.)

The relative flight cost ratio 〈*P*_b2,3_〉/〈*P*_b1_〉 can be expressed either in terms of *ω*/*ω*_0_ or *A*/*B* and *ω*/*ω*_c_:3.25
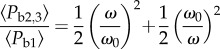
and3.26
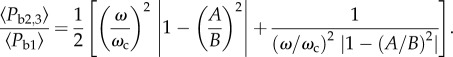
A colour-coded contour plot of this function is presented in figure 7 (online).

## 4. Non-quadrature phase

**Figure 8.**
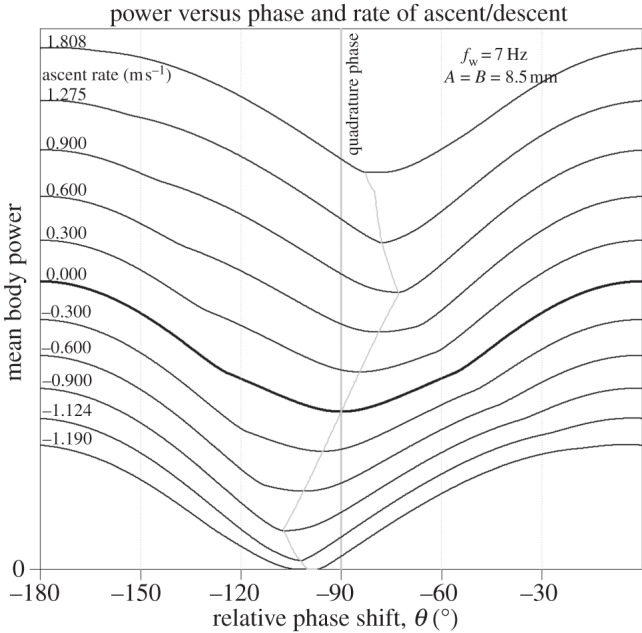
When the bird is ascending or descending, the quadrature phase arrangement is not always optimal. However, the phase *θ* for which the power is minimized only deviates significantly from −90° during extreme rates of ascent or descent. The results here simulate a bird with a wingstroke frequency, *f*_w_ = 7 Hz and equal amplitude oscillations on both forward and vertical axes of amplitude 8.5 mm. The locus of points at which the power is minimized at climb rates intermediate between those of the marked traces is also shown.

We reiterate that this modelling pertains to steady, horizontal flight. The possibility exists that departures from quadrature phase may be advantageous during ascent or descent, but the mathematics in such cases is considerably more involved. Nevertheless, these situations were numerically investigated under the assumption that *A* = *B* with an additional power component representing the change in gravitational energy with time, whose mean value can be estimated using an altimeter or GPS device [34,35]. The results are presented in figure 8. It can be seen that predictions of the quadrature phase model are still accurately upheld in most circumstances involving realistic rates of ascent or descent.

**Figure 9.**
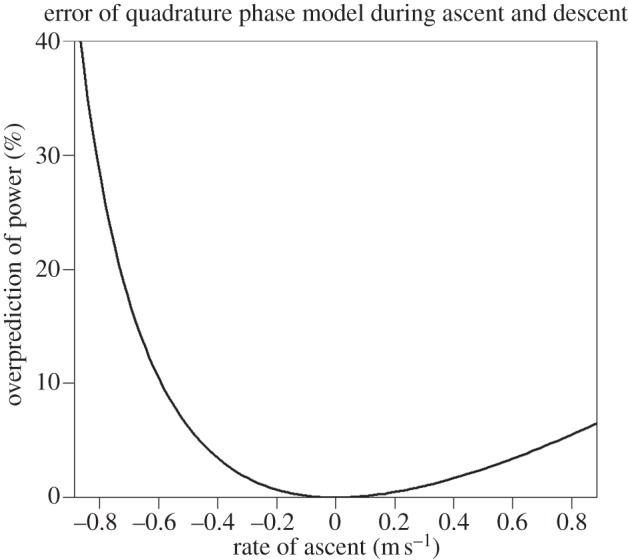
The quadrature phase assumption potentially overpredicts the body power for ascending or descending flight. The error is plotted here for climb rates within the range ±0.9 m s^−1^, where the optimum phase appears to deviate linearly from *θ* = −90° in figure 8. The model is relatively accurate for sustainable rates of ascent but errors grow rapidly for high rates of descent.

Although the optimal arrangement is never far from quadrature phase, the error is sensitive to the rate of ascent, and is asymmetrical in that it grows faster with descent than ascent. This is evident from the plot presented in figure 9. It has been reported that the power requirements of moderate ascending and descending flight in pigeons can be accurately estimated by summing the power required for level flight with the rate of change of gravitational potential [36]. Interestingly, the same study found that a discrepancy did arise for high descent rates but not for high climb rates, descent being clearly more expensive than anticipated. When flying steeply downwards at a descent angle of −60°, the pigeons flew at a horizontal velocity of 3.6 m s^−1^ and a vertical rate of descent exceeding 3 m s^−1^. Hence, one possible explanation for this could be that flight costs during rapid descent are so minor and so rarely encountered that there is little need for birds to acquire biomechanical flexibility that would allow significant deviations from quadrature phase. However, this descent rate is more rapid than that which would be expected if the bird were simply gliding (typically no more than 2 m s^−1^ for a pigeon).

If more accurate estimates of biomechanical power are required then direct integration is an option, obtaining relative velocities and displacements for the forward and vertical axes from which the individual energy terms can be derived, combining this information with altitude data (if available) to obtain body energy, and calculating power by numerically evaluating the mean rate of energy increase with time, being careful to ignore periods when the total energy of the bird is decreasing. This approach may offer improved precision, particularly when power is not all concentrated at the fundamental wingstroke frequency or when large departures from quadrature phase are expected.

## 5. Discussion

Despite the considerable complexities involved in flapping flight, by focusing on the consequences for the mechanical motions of the body, this oscillatory energy fluctuation model provides a useful initial step in theoretically underpinning the use of body-mounted accelerometers to estimate the relative costs of horizontal flapping flight in birds or bats. Acceleration-based proxies for the power detectable in the body of an animal during flight have been derived from first principles ((3.5), (3.7) and (3.9)) and in the appropriate units of ML^2^T^−3^. Within these equations, the DBA formalism is encapsulated very naturally via r.m.s. acceleration ((3.14) and (3.15)), or aRMS. These expressions represent a substantial improvement over attempting to estimate the biomechanical energy expenditure or metabolic rate of flying animals from ODBA or VeDBA alone. The magnitude of ODBA varies with orientation, and different rescaling factors must be applied to single axis projections of ODBA and VeDBA if they are to be used to estimate 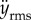 or 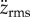 (appendix B). We point out that aRMS, which is more closely related to VeDBA than ODBA, is just as straightforward to calculate. Furthermore, aRMS may be more universally applicable in future mathematical and empirical studies of animal locomotion. DBA measures lack the units of power and so will always require a direct calibration against the rate of energy turnover. Their use in estimating the biomechanical costs of flapping flight has generally lacked a firm theoretical basis, pays no heed to sensor orientation relative to gravity, ascribes undue significance to accelerations in the horizontal plane and overlooks the significance of wingstroke frequency. As can be seen in (3.22), body power is rather sensitive at times to wingstroke frequency, so may in itself provide a valuable means of gauging flight effort independently of body power estimation.

This analysis furnishes several novel parameters that may provide insights into the kinematics of flight. In particular, it predicts that there may be two regimes of flight with regard to power production and wingstroke frequency and that there may be a transition from slow to moderate intensity flight, when equation (3.23) and figures 6 and 7 suggest power savings may sometimes be possible. For steady horizontal flight involving sinusoidal vibrations on the forward and vertical axes, it has been shown that a quadrature phase arrangement is potentially advantageous. If flight strategies could exploit this, temporarily storing and retrieving energy during each wingbeat cycle, it might be possible for some species to avoid or curb what would otherwise be a third-order sensitivity in mean body power to wingstroke frequency. Due to the ubiquity of predators, the need to catch airborne prey and the general requirement for economy of locomotion, there may have been considerable evolutionary pressure for birds to waste very little energy at wingstroke frequencies exceeding *ω*_0_. Nevertheless, body-mounted accelerometry is not privy to the subtleties of wing flexion, angle of attack and feathering, so this model leaves open the possibility that birds have considerable scope to adjust their flight style without necessarily compromising efficiency.

When birds are flying in the linear regime then equation (3.14), which reflects the costs of combating gravity, shows that only the vertical, gravity-aligned component of the acceleration should enter into the calculation of body power. Although forward accelerations become relevant to power estimation for the asymptotically cubic regime, equation (3.15) shows that their contribution to body power is *subtractive*. This somewhat counterintuitive need to subtract 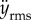 from 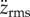 in (3.7) stems from the fact that variations of gravitational energy do not always mask variations in kinetic energy. However, quadrature oscillations in *K_y_* tend to erase fluctuations in *K_yz_*, not reinforce them.

While the  model directly considers vibrations only on the body of the bird, owing to mechanical coupling, the same kind of oscillations and trade-offs should also apply to the horizontal and kinetic components of the wings. However, because the centre of mass of the wings must travel a much greater distance during each wingstroke than the centre of mass of the body, for any given wingstroke frequency, fluctuations in wing kinetic energy grow quadratically with wingstroke excursion, but variations in wing gravitational potential grow only linearly. Therefore, the onset of the transition between the linear and asymptotically cubic flight power regimes might be generally expected to occur at a lower frequency for the wings than for the body. The kinematics of the wings, which cannot be directly measured by the accelerometer, should generally dominate the overall biomechanical costs of flight. Indeed, kinetic energy fluctuations might easily become a more important consideration than compensating for the gravitational energy losses of each wingbeat. If the gravity terms are neglected, then (3.7) and (3.9) simplify somewhat and predict 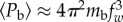 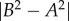. This could be especially true of energetic high-speed flight, even though there may then be significant wing retraction and supination to avoid undue aerodynamic drag [37,38]. Because the forces involved in wing retraction tend to cancel on the body due to bilateral symmetry, the costs are hidden from body-mounted accelerometers. Hence, the ratio of perceived body power to true total biomechanical power might be somewhat reduced during intense flight for some species, which may well require the tailoring of flight models to each species in the future, following empirical observations and extensions of the modelling.

A number of other original summary statistics could prove useful to the interpretation of body-mounted accelerometry data obtained from flying birds. The ratio *A*/*B* may help to characterize the mode of flight performance and perhaps also evaluate the skill and dexterity of individual birds. With the possible exception of hovering flight when birds may be able to recoup some of the kinetic energy stored in the air during the previous half-stroke, flying animals generally have no means of recovering energy lost to the environment. However, vibrations on the forward axis offer convenient energy storage which may also be exploited to reduce pitching of the body. Many birds use various reflex mechanisms during flight to subdue head vibrations and thereby avoid vision impairment [33]. Body accelerations have a total amplitude 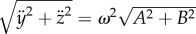 which for small *A*/*B* can be approximated by *ω*^2^*B*(1 + *A*^2^/2*B*^2^). Hence, the additional loss of visual acuity due to a small *A*/*B* ratio would be relatively imperceptible, implying little need for *A*/*B* → 0.

The ratio *A*/*B* may also be particularly sensitive to effort in realistic situations, correlating with wingstroke frequency and increasing at higher forward velocities. The relative phase lag *θ* between the vertical and forward axes may act as a marker of ascending or descending flight, or reflect efforts to synchronize wingstroke frequency with other birds during V-formation flight. We also expect the ratios *ω*/*ω*_c_, *ω*/*ω*_opt_ and *ω*/*ω*_0_ to be informative regarding flight intensity and flight efficiency. Collectively, these measures may also crudely encode hints as to the altitude at which the bird is flying. Due to the complexities of wing kinematics and anatomical constraints, it is very likely that no simple unifying pattern will adequately summarize all species, but departures from normality are often the most interesting aspects of biological research and so additional parameters can prove very useful in highlighting departures from non-conformity. Therefore, these flight variables may be particularly valuable in helping to unpick the challenges involved in flying efficiently. The static acceleration also offers a potentially illuminating variable for flying animals which has been largely ignored to date. Birds can sustain prolonged banking when circling or jostling for position within a cluster flock [39], and any drift in the mean direction of the momentum vector induces a non-gravitational contribution to the static acceleration. Thus, when the static acceleration deviates appreciably from gravity, it would suggest that the bird is not undergoing steady horizontal flight. However, the converse is not true, because one also expects the static acceleration to tally with gravity during steady non-banking ascending or descending flight. Therefore, the distribution and time variability of the static acceleration can be informative.

Dimensional considerations may allow the results obtained here to be extrapolated to some degree, particularly regarding the estimation of biomechanical power from accelerometry for aquatic animals. Due to the buoyancy afforded by water, the estimation of inertial costs for aquatic species during swimming is not encumbered by gravitational considerations [2]. This invulnerability to gravity suggests that the cost of swimming should correlate with the product of body mass, the period of the swimming stroke and some function of the square of the decomposed r.m.s. accelerations, dependent upon the anatomy of the species under consideration. Locomotion costs in terrestrial animals are likely to be more complex: weight support can either be provided continuously or episodically by the ground.

Naturally, there are limitations to what a body-mounted accelerometer alone can glean about flight. During free-ranging flights, there could be circumstances where basic inferences may be misleading, particularly if the rate of ascent or descent is unknown. In addition, special care may be needed when attempting to disentangle the static and dynamic accelerations for birds using intermittent modes of flight such as flap-gliding or flap-bounding. Accelerometers cannot infer absolute velocities in any direction, and many birds exploit the assistance of thermals, following winds and airflow over uneven terrain, all of which are capable of drastically altering the power requirements of flight. Nevertheless, the present model offers a practical and non-invasive method of extracting from accelerometry a variety of parameters that could be informative concerning flight style and performance, while also providing an explicit procedure for determining biomechanical body power in free-flying birds which may be generally proportional to overall flight power. In the complex processes that transform the biochemical energy of birds into atmospheric vortices, turbulence and heat, aerodynamic costs lie downstream of the biomechanical costs. While it might eventually be possible to incorporate them within an extended model, the formidable challenges of contending with complicated wake patterns, vortex interactions and chaotic flow patterns continue to plague theoretical models, and quasi-static approximations to the Navier–Stokes equations commonly used in aerodynamic analysis inherently limit their accuracy and usefulness [40]. A more realistic near-term goal would be to broaden the present modelling to include wing kinematics and morphology. This will inevitably necessitate the input of anatomical information allowing the body power relationship to accommodate allometric differences between species and also address wing-propelled locomotion in the media of differing densities. Due to the intrinsic complexities, we anticipate that experimental data collected from a variety of species will be required. Birds are graceful aeronauts, skilfully adjusting their posture and technique in ways we have only started to perceive [41]. However, a battery of physiological, biomechanical and aerodynamic techniques can augment and refine one another when quantifying flight costs. These complementary approaches to studying avian energetics hold much promise in arriving at a more unified understanding of the compromises involved when animals fly—whether they are foraging for food, migrating, chasing airborne quarry or evading predators.

## Figures and Tables

**Figure 10.**
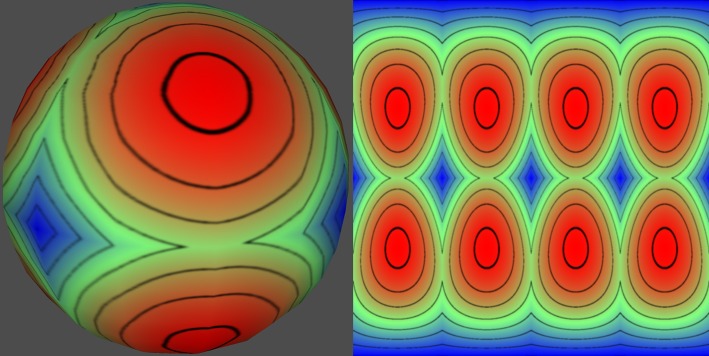
Variation of the error in ODBA with orientation, rising from a minimum of zero (six centres of the square contours) to a maximum of 73.2% (eight centres of the circular contours) with a mean error of 50% (contours at 10% intervals). A single contiguous contour is obtained when ODBA/VeDBA = 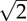. (Online version in colour.)

## Appendix A. Reorientation during post-processing

When flying in a straight line, the static acceleration , where  represents the time-averaged mean of the *k*th accelerometer channel, should equal the gravitational acceleration, ***g****.* When this does not hold, it is possible that the bird is turning, undulating, bounding or flying through zones containing vertical air currents. Centripetal acceleration combines with gravity during turns, the direction of the resultant vector determining the degree of banking necessary in order that the bird experiences the net acceleration dorsally so that the forces on the wings are symmetrically balanced. At such times, the increase in the static acceleration relative to gravity is a useful guideline as to the departure from linear motion. For uniform rectilinear motion, the mode of the distribution of the static acceleration magnitude can be identified with gravity. To some degree, this allows for the self-calibration of accelerometry data.

When ||***a****_s_*|| ≈ ||***g***||, a condition that is easily checked, it is straightforward to calculate the vertically aligned component of the acceleration *a*_vert_ using the scalar projection of ***a*** onto ***a****_s_*:

A1

This vertical acceleration is the combination of both static and dynamic components, so the dynamic vertical acceleration *a_z_* can be obtained by subtracting the time-averaged value of *a*_vert_ using

A2

Projections of ***a*** orthogonal to the vertical axis will then lie in the horizontal plane:

A3

and

A4

As before, the dynamic acceleration is obtained by subtracting the static acceleration ( and ). A means of determining *a_x_* and *a_y_* from *a*_h1_ and *a*_h2_ is then required. By symmetry, one expects the dynamic acceleration of the forward (*y*) axis to exceed that on the lateral (*x*) axis. One way to proceed would be to first determine the fundamental wingstroke frequency using *a_z_* then bandpass filter *a*_h1_ and *a*_h2_ using fast Fourier transforms so that only frequencies near the fundamental wingstroke frequency are retained. Following this, one could compute the angles *ϕ* = arctan(*a*_h__2_/*a*_h__1_), compile a circular histogram of the results and use the angle corresponding to the peak in the histogram *Φ*, to reorient *a*_h1_ and *a*_h2_ as follows:

A5

and

A6

There is a potential ambiguity in this result concerning the polarities of *a_x_* and *a_y_*. It arises due to the fact that one expects two peaks in the angular histogram separated by *π*. Therefore, one should also evaluate *a_x_* and *a_y_* using *Φ* ← *Φ* + *π*. In practice, there may be no need to calculate *a_x_* because it is unlikely to represent interesting information, but the polarity of *a_y_* is potentially important if one is eager to know, for example, how the phase shift between the forward and vertical axes varies with time. This model expects that the displacement on the *y*-axis will always lag behind that of the *z*-axis, and hence *a_z_* should always lead *a_y_*, resolving the ambiguity. One can again apply bandpass filtering to *a_y_* and *a_z_* around the detected wingstroke frequency in order to test which value of *Φ* is appropriate. The phase shift can be accurately measured in the recovered time domain after bandpass filtering using linear interpolation between samples in the vicinity of the positive/negative going zero crossings. If the orientation of the accelerometer is fixed with respect to the body of the bird, then this process need only be performed once, and the value of *Φ* can then be reused without recalculation.

## Appendix B. Relationship between overall dynamic body acceleration, vectorial dynamic body acceleration and r.m.s. acceleration

Consider the instantaneous dynamic acceleration ***a***_d_ = (*a_x_*, *a_y_*, *a*_z_) derived from a triaxial accelerometer. The generalized mean or *L^p^*-norm of the components of ***a***_d_ is defined as

B1

Therefore, the magnitude of ***a***_d_ according to VeDBA is  whereas its magnitude according to ODBA is |*a_x_*| + |*a_y_*| + |*a_z_*|. Converting to spherical coordinates (*r*, *θ*, *ϕ*), one has

B2

B3

B4

The magnitude of ***a***_d_ using VeDBA is thus

B5

This confirms the standard expectation of Euclidean trigonometry and the Pythagorean theorem. However, the magnitude of ***a***_d_ in the case of ODBA generally disagrees with this because

B6

and

B7

Due to the second term, ODBA will exceed VeDBA unless at least two of the three acceleration components are zero. The two will agree only when there is alignment of the acceleration with one of the three measurement axes. Hence, the response of ODBA varies according to orientation.

To find the maximum error in ODBA, let *a_y_* = *a_x_* + *α* and *a_z_* = *a_x_* + *β* where, without loss of generality, it can be assumed that neither *a_x_*, *a_y_* nor *a_z_* are negative. Now let ζ = (ODBA^2^/VeDBA^2^ − 1)/2 so that the stationary points of ζ will be identically located to those of ODBA/VeDBA:

B8

Equating to zero the partial derivatives of *ζ* with respect to *α* and *β* gives

B9

and

B10

These expressions reduce to

B11

and

B12

The first condition is satisfied only if *α* = *β*, which also ensures the second is satisfied. When *α* ≠ *β*, the second condition holds if *α* + *β* = −3*a_x_* which cannot be true given that neither *a_y_* nor *a_z_* are negative, which implies that *α* + *β* ≥ −2*a_x_*. This leaves only *α* = *β* and hence |*a_y_*| = |*a_z_*| after restoring moduli. For |*a_x_*| → 0, one then finds that (ODBA/VeDBA)^2^ can be no less than 2. This can be seen by letting *γ* = |*a_y_*| = |*a_z_*| and |*a_x_*| = *ε* with :

B13

However, as |*a_x_*| → |*a_y_*| = |*a_z_*| one finds that (ODBA/VeDBA)^2^ can be no greater than 3. This can be seen by letting |*a_x_*| = *γ* ± *ε* where, once again,  and *γ* = |*a_y_*| = |*a_z_*|:

B14

Hence, ODBA/VeDBA is maximized when the acceleration components on each axis have an identical magnitude. Because the minimum is already known to occur when two of the components are zero, one may conclude that ODBA is confined to the range

B15

It is interesting to ask whether VeDBA can be recovered from historical records of ODBA. This is possible only in an approximate statistical sense, and best results would be obtained when data have been collected from animals whose orientation in space varies considerably or in situations where the orientation of the accelerometer itself is free to drift. In order to determine the rescaling factor, it is first necessary to calculate the mean exaggeration of ODBA relative to the true acceleration magnitude. Due to symmetry, it is sufficient to consider the solid angle *Ω* = *π*/2 corresponding to the octant 0 ≤ *θ* ≤ *π*/2, 0 ≤ *ϕ* ≤ *π*/2. Although the mean value of VeDBA is simply the vector length *r*, the mean value of ODBA is

B16

B17

B18

B19

B20

Therefore, on average, ODBA exaggerates the true acceleration magnitude by 50% and so, in some circumstances, ODBA data records can be translated into estimates of VeDBA simply using

B21

A highly significant linear relationship between these two measures has already been experimentally observed, with the best fit corresponding to VeDBA ≈ 0.014 + 0.6418 ODBA for units of ***g*** [27]. The slope of this empirical relationship agrees with the theoretical value to within 4%. In the same work, the envelope of figure 2 exhibits a wedge distribution whose upper and lower slopes are approximately unity and , corresponding to the anticipated range in error of ODBA due to changes in orientation.

When evaluating r.m.s. accelerations, there is no explicit requirement to calculate vector lengths, but we stress that the Euclidean formulation, as adopted by VeDBA, is implicit in the present modelling. While VeDBA and r.m.s. acceleration are in agreement here, and one would generally expect to find an excellent correlation between them, their magnitudes are anticipated to differ whenever there is any spread in the distribution of the dynamic acceleration data. For complex or aperiodic acceleration profiles, we recommend the r.m.s. method over VeDBA in all cases, because the ambiguity implies that no single rescaling factor will suffice. However, for sinusoidal motion *ψ* = *β* sin(*t*) along a straight line, a simple rescaling is possible. The time-averaging used by VeDBA follows that of ODBA, namely the *L*^1^-norm or arithmetic mean. However, that of the r.m.s. prescription follows the *L*^2^-norm or quadratic mean which, as found previously in (3.11), yields . If follows from the properties of the generalized means that  will never be smaller than  and its value is

B22

For this simplified situation, one finds   and hence

B23

In applications where approximate alignment of the accelerometer's *z*-axis with gravity is attempted, small changes in orientation occurring at frequencies that are not rejected by the sampling window will cause ODBA to increase even in the absence of vibration. Because the magnitudes of *a_x_* and *a_y_* are then much smaller than gravity one finds that

B24

Hence, the lack of rotational invariance in ODBA (as depicted in figure 10) then causes it to respond linearly to tilting of the accelerometer, tending to accentuate the sensitivity of ODBA to activity. Dependent on the moment of inertia there can be a cost associated with periodic adjustments in attitude, as might occur in animals exercising on a treadmill, and ODBA may be sensitive to it. For arbitrary orientations with respect to gravity, ODBA can both exaggerate and underestimate changes in the acceleration magnitude caused by rotation. Almost any acceleration measure is vulnerable to errors when the rotation of a transducer is not limited to low frequencies because it is then impossible to accurately separate the static and dynamic accelerations without information from a gyroscope.

When significant rotation exists, in the absence of a gyroscope, some measure of the variability of the instantaneous magnitude of the total acceleration vector ***a*** could offer an alternative proxy for biomechanical effort. For instance, one might calculate the standard deviation of  for all the raw outputs of the accelerometer falling within some time interval. This could prove informative whenever a significant component of animal activity involves body rotation. Because accelerometers remain sensitive to gravity even within buoyant media, this may be especially useful in the context of aquatic animals as they are not constrained by gravity when swimming underwater. In circumstances where the dynamic acceleration is extremely small, activity can also be estimated by quantifying the rate of body reorientation d*ϕ*/d*t*. This can be approximated as *Δ**ϕ*/*Δ**t* using pairs of static acceleration vectors  and  separated by a fixed time interval *Δ**t* appropriate to the rotation rates of interest:

B25

and

B26
